# The culture of radiology: Learning, teaching and curiosity

**DOI:** 10.4102/sajr.v24i1.2045

**Published:** 2020-12-21

**Authors:** Maya Patel

**Affiliations:** 1 Department of Radiology, School of Medicine, Faculty of Health Sciences, University of the Witwatersrand, Johannesburg, South Africa; 2 National Radiology Services Inc, Johannesburg, South Africa

Radiology is a world interposed between macroscopy and microscopy, capturing images that afford clinicians and surgeons with an in-depth analysis of the interior of the human body. It is a core instrument in their armamentarium for medical practice, analogous to the body’s heart, lungs, brain and spine.

Revolutionary technological advances are ensuring rapid enrichment in the discipline of radiology from image enhancement and resolution to faster acquisitions, improved patient comfort and artificial intelligence (AI). The relentless strive to raise the bar from competing technicians and engineers is creating a speciality that is becoming a challenge to foster and maintain academically, particularly when access to these newer strategies is limited by cost and availability.

Staying on par with these advances means that with each passing year, new learning paradigms are necessary. Within our environment, this includes sub-specialisation, incorporation of AI and different teaching techniques and research.

Sub-specialisation focuses expertise in one area of radiology, providing clinicians with well-informed opinions and interpretations and ultimately a better outcome for the patient. Whilst this is well established in the medical and surgical fields, radiology is trailing behind, particularly in developing countries with limited resources and lack of established protocols for specialisation. Although certain areas in radiology have already dichotomised, such as paediatric radiology, musculoskeletal radiology and mammography, on the whole, radiology in South Africa is still dominated by general radiologists. Given the wide range of health service delivery needs from rural to urban areas and the high burden of trauma surgery and emergency medicine, the role of the general radiologist is pivotal to our healthcare system. We must rise to meet this public health need, but at the same time, not ignore the equally demanding need to join our subspecialist colleagues and ascend the steep learning curve. Thankfully, international courses, fellowships, webinars and online resources are plentiful, and the path towards fixating on a niche is now more accessible. Given the lack of established registered local university programmes currently, the onus lies with the radiologist to pursue such avenues.

Our next call is to embrace AI in radiology. Welcome or not, AI is steadily permeating its way into our lives. The promise of ease of use, objective results, saving time, reproducibility and digital twins is far too alluring for both the clinician and the patient. Although it is more likely to become embedded into daily practice in resource-rich, high expenditure countries because of cost, this amalgamated form of practice in radiology will probably be more useful in high disease burden regions such as ours. We need to ensure constant growth in information technology infrastructure and accessibility to these inventions. The role of AI should remain supportive, and it is up to the radiologist to ensure this. A lesson from the coronavirus pandemic includes exploring different ways of interacting with others in a ‘safer’ manner and AI offers such an opportunity.

A further test of our performance is related to research in the field of radiology. We need to awaken our curiosity and ask more questions, given that we are in a country that offers a wealth of opportunity with the disease burden we face. Research is the third category in the field of learning, together with an expert faculty of teachers and written learning material, the model upon which most medical schools and universities are developed. Whilst germination has begun, radiological research in South Africa still requires a fair amount of incentive, cultivation and grooming. Here again, a local structured model is lacking and there is a hiatus for new prospects, which we need to embrace. This duty rests not only with the higher educational institutions but with all of us who can contribute to sharing new knowledge and expanding our field with what we have to offer.

The universal future for radiology involves life-long training, growth and adaptability. In particular, the South African radiologist needs to foster a culture of learning, teaching and curiosity. By working together, this will ensure that we expand our intellectual environment, provide the best opinions for our patients and practice safe radiology. The future is in our hands – are you prepared?

*‘Never stop learning because life never stops teaching*’ … Anonymous

Dr Maya Patel

